# Prognostic Factors and Quality of Life in Vulvar Cancer Patients: 12-Year Results from a Eastern European Center

**DOI:** 10.3390/jpm15070266

**Published:** 2025-06-22

**Authors:** Mihai Stanca, Henrietta Becze, Alexandra-Maria Pop, Dan Mihai Căpîlna, Szilard Leo Kiss, Cristian-Ioan Cîmpian, Mihai Emil Căpîlna

**Affiliations:** 1 First Obstetrics and Gynecology Clinic, George Emil Palade University of Medicine, Pharmacy, Science, and Technology of Targu Mures, Gheorghe Marinescu Street, Number 38, 540142 Târgu Mureș, Romania; 2 Faculty of Medicine, George Emil Palade University of Medicine, Pharmacy, Science, and Technology of Targu Mures, Gheorghe Marinescu Street, Number 38, 540142 Târgu Mureș, Romania

**Keywords:** vulvar cancer, prognostic factors, quality of life, surgery, adjuvant therapy

## Abstract

**Objectives:** Despite the relatively high incidence of vulvar cancer, there is a noticeable lack of studies in Romania and other Eastern European countries focused on evaluating the long-term oncological outcomes and Quality of Life (QoL) for patients with this condition. **Methods:** A total of 91 patients were included in the study. The first objective was to evaluate the 5-year overall survival (OS) in patients with vulvar cancer at International Federation of Gynecology and Obstetrics (FIGO) stages IA-IVA who underwent surgery, ±adjuvant radiotherapy (RT). Additionally, the study aimed to identify prognostic factors that could either positively or negatively influence survival outcomes in these patients. The second objective was to assess the QoL, conducted using validated questionnaires issued by the European Organization for Research and Treatment of Cancer, specifically the QLQ-CX30 and QLQ-VU34. **Results:** The patients had an average age of 67.7 years (38–91). At the time of assessment, 51.6% of the patients were alive. Additionally, the 5-year OS was reported at 45%. The multivariate analysis indicated that age ≤ 50 years (*p* < 0.03), FIGO stage IB (*p* < 0.007), and tumor differentiation grade I (*p* < 0.01) were associated with improved survival rates. Conversely, age > 80 years (*p* < 0.05), FIGO stages IIIB (*p* < 0.01) and IIIC (*p* < 0.06), tumor size > 5 cm (*p* < 0.02), positive resection margins (*p* < 0.03), lymph node metastasis (*p* < 0.06), and pelvic exenteration (*p* < 0.002) were identified as independent negative prognostic factors. Of the 47 living patients, 32 completed the QoL questionnaires. The respondents reported a decent overall QoL score of 65.3. However, treatment-specific symptoms, such as vulvar scarring, vulvar swelling, groin lymphedema, and leg lymphedema, had a negative impact on QoL. Consequently, functional symptoms like fatigue, pain, and sleep disturbances persisted, leading to a body image perception score of 33.7 on a scale from 0 to 100. **Conclusions:** This study highlights decent OS and QoL outcomes. It is important to note that vulvar cancer primarily affects older women. In this study, 51.6% of patients were over 70 years old at the time of surgery. Consequently, the 5-year OS of 45% could not be attributed solely to oncological factors, as most of these patients did not die from recurrences but rather from associated comorbidities. The findings of this study provide a foundation for future randomized controlled trials aimed at further enhancing vulvar cancer patients’ care and outcomes.

## Simple Summary

Romania lacks comprehensive statistical data on vulvar cancer and has not been included in large multicentric European studies evaluating prognostic factors and Quality of Life for this condition. This creates a pressing need for research, especially in Romania and other Eastern European countries where patients often do not receive adequate government support. The findings of this study will lay the groundwork for future randomized controlled trials.

## 1. Introduction

Vulvar cancer is the fourth most common gynecological cancer, accounting for 5–8% of all gynecological malignancies, typically affecting postmenopausal women [1]. Approximately 65% of these cases are found in high-income countries [2]. According to the latest Globocan statistics, the global incidence of vulvar cancer is estimated at 45 cases per 100,000 women, with a mortality rate of 17.4 deaths per 100,000 women [3,4]. In Romania, the prevalence rate stands at 14.4 cases per 100,000 women [5]. Vulvar cancer can develop via two primary pathways: one associated with human papillomavirus (HPV) and another that occurs independently of HPV. The HPV-independent pathway is more commonly observed in older women [6]. HPV infection can result in the development of precancerous lesions, and if left untreated, approximately 80% can progress to invasive disease [7]. Other risk factors for the development of vulvar cancer include advancing age, cigarette smoking, inflammatory disorders affecting the vulva, and compromised immune function [6].

Squamous cell carcinomas make up 90% of all cancerous vulvar tumors, with about 50% of these cases asymptomatic [8]. According to the SEER database [6], 5-year overall survival (OS) rates differ by International Federation of Gynecology and Obstetrics [9] (FIGO) stage: 86% for localized disease (stages I–II), 57% for regional or locally advanced disease (stages III–IVA), and 17% for distant metastasis (stage IVB) [6]. In many higher-income countries, when measured, the estimated 5-year OS is about 50% to 70% [2,10].

The treatment approach is determined by the histological characteristics of the disease, its stage, and the patient’s overall performance status. This may include options such as surgery, chemotherapy, radiotherapy, and supportive care aimed at palliative management [11]. Patients diagnosed at an early stage of the disease are typically treated with either a wide local excision or a modified radical vulvectomy, which may include unilateral or bilateral sentinel lymph node biopsies (SLNB) or inguinofemoral lymphadenectomy [12,13]. The latter mentioned is often needed, particularly in cases where the tumor exceeds 4 cm, multiple invasive lesions are present, or there is a suspicion of inguinal lymph node involvement based on clinical or radiological findings [4,14]. A complete inguinofemoral lymphadenectomy involves the surgical excision of all lymph node-containing fatty tissue located in the superficial inguinal and deep femoral regions, specifically medial to the fossa ovalis [15]. However, approximately 30% of patients present with locally advanced disease that has spread to nearby organs and/or lymph nodes (LNs) [16]. The key risk factors associated with nodal metastasis include clinical nodal status, age, differentiation grade, tumor stage and size, the depth of stromal invasion, and the occurrence of lymphovascular space invasion (LVSI) [17]. In contrast, evidence on recurrence patterns in vulvar cancer is scarce, with most recurrences typically occurring within the vulvar area [8]. For these individuals, more extensive procedures, such as pelvic exenteration (PE), may be required to achieve clear resection margins. In cases of recurrent vulvar cancer, particularly after initial chemoradiation, PE remains the only treatment option that could provide a curative chance for select patients whose disease has spread to pelvic organs but not to distant sites [12].

This study aims to achieve two primary objectives. The first objective is to assess the prognostic factors that influence the 5-year OS in patients with vulvar cancer stages FIGO 2021 [9] IA-IVA undergoing surgery ± adjuvant radiation therapy (RT). The second objective is to evaluate the Quality of Life (QoL) of survivor patients through standardized questionnaires issued by the European Organization for Cancer Research and Treatment (EORTC), specifically the Quality of Life Questionnaires-QLQ-C30 [18] and QLQ-VU34 [19,20].

This study is necessary due to the absence of comparative research in Romania and other Eastern European countries. Although there are substantial studies on this topic in Central, Western, and Northern Europe, as well as in the USA and China, similar research is notably lacking in Romania and the broader Eastern European region. The only comparable study in Eastern Europe was conducted in Croatia in 2021 by Miljanovic-Spika et al. [7], which focused on prognostic factors in vulvar cancer but did not assess patients’ QoL.

## 2. Materials and Methods

This study received approval from our Institute’s Ethics Committee (approval code: 27499, 18 November 2024). Additionally, we obtained permission from the EORTC to use their QoL questionnaires, specifically the QLQ-VU34 [19,20] and QLQ-C30 [18]. All participating patients provided informed written consent.

### 2.1. Study Design and Patient Population

This retrospective observational study included 91 patients who met the inclusion criteria, treated and followed up at the First Obstetrics and Gynecology Clinic of Târgu Mureș, Romania. The authors analyzed the demographic data, clinical details, diagnosis, staging, treatment methods, disease outcomes, and survival. Patients were evaluated by a multidisciplinary team consisting of experienced gynecologic oncologists, medical oncologists, radiotherapists, pathologists, radiologists, and anesthesiologists. Each patient underwent a series of essential clinical, hematological, imaging, and pathological assessments. For select individuals, imaging techniques such as computed tomography (CT) scans, magnetic resonance imaging (MRI), and cysto-sigmoidoscopy were recommended.

Disease staging was determined following the FIGO 2021 classification system [9]. Treatment plans were tailored based on the disease stage, histological type, performance status, and the possibility of achieving a tumor-free resection (R0). Regular follow-ups post-treatment were advised in accordance with ESGO guidelines [4]—every three months during the first year, followed by six-month intervals until the fifth year, and then annually thereafter.

### 2.2. Data Assesment and Statistical Analysis

Statistical analysis involved descriptive statistics (frequency, percentage, mean, median, standard deviation) and inferential statistics. The Shapiro–Wilk test assessed data distribution. The Mann–Whitney test was used for comparing two non-Gaussian data sets, while the Kruskal–Wallis test, along with Dunn’s test for multiple comparisons, was used for more than two sets. The Chi-square test evaluated associations between qualitative variables. To examine the impact of multiple variables on event timing, univariable and multivariable Cox regression analyses with hazard ratio were performed, and Kaplan–Meier curves were analyzed for survival. A significance threshold of *p* < 0.05 was established. Analysis was conducted using SPSS version 29.0 (SPSS, Chicago, IL, USA). The lack of a prospective power analysis suggests that the study might be underpowered to detect small to moderate effect sizes. Therefore, the results should be interpreted with caution, and further research with larger, prospectively designed studies is needed to confirm these findings.

### 2.3. Inclusion and Exclusion Criteria

The study included patients with either recurrent or non-recurrent vulvar cancer, confirmed through clinical evaluation, histopathology, and imaging techniques, and categorized according to the revised FIGO 2021 stages IA-IVA [1,9]. These patients underwent surgery for vulvar cancer, with or without adjuvant RT. Other types of therapies were excluded due to their limited prevalence and potential to influence the results, which were not expected to yield statistically significant outcomes. Furthermore, patients with distant metastases or synchronous cancers were also excluded (see Table 1 and Table 2).

In terms of QoL assessment, only patients who had no recurrence, no known comorbidities, and agreed to participate in the study were included. Additionally, only those with a known follow-up were considered eligible. The use of complete case analysis in the QoL assessment may have reduced the sample size and statistical power, potentially limiting the generalizability of the findings.

### 2.4. Treatment Administered

The patients received the following treatments: Wide local excision, which involves the removal of the area containing the lesion, along with a margin of healthy tissue surrounding it. This procedure is recommended for small tumors and for older patients; hemivulvectomy (anterior, posterior, left or right), which involves the removal of a portion of the vulva along with adjacent structures; and total radical vulvectomy, which entails the complete removal of the vulva along with the surrounding soft tissues.

In cases of urethral invasion, up to 2 cm of the distal urethra was resected without significantly affecting urinary continence. Additionally, in some patients, a distal colpectomy was performed to ensure clear resection margins.

PE involved the removal of all pelvic organs, including the vulva, vagina, cervix, uterus, and bladder ± rectum, including the need for inguinofemoral and/or pelvic lymphadenectomy.

**Figure 1:**
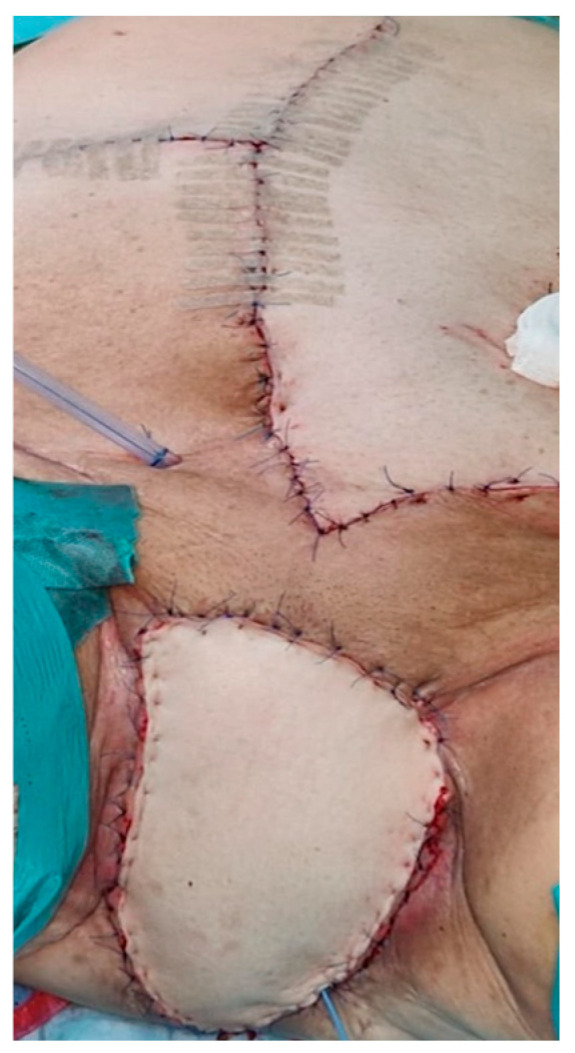
Vertical rectus abdominis myocutaneous (VRAM) flap in a patient with PE.

In some patients, the perineal defect was successfully covered using a V-Y flap, a rhomboid flap, or a vertical rectus abdominis myocutaneous (VRAM) flap (Figure 1).

In relation to lymphadenectomy, sentinel lymph node biopsy could not be performed due to a lack of logistical support, despite the latest recommendations [4,21]. Consequently, the approach to lymphadenectomy was determined by the proximity of the tumor margin to the median line. If the tumor margin was less than 2 cm from the median line, a unilateral inguinal–femoral lymphadenectomy was performed. In cases where the tumor margin exceeded 2 cm from the median line, a bilateral inguinal–femoral lymphadenectomy was carried out.

Deep inguinal–femoral lymphadenectomy was conducted, beneath the cribriform fascia.

Adjuvant RT was administered following the final histopathological examination, which revealed positive resection margins and LN metastases.

### 2.5. Quality of Life Questionnaires-QLQ-C30 and QLQ-VU34

To all alive and eligible patients who met the inclusion criteria, a postal letter was sent that included an informed consent form and a set of validated self-administered questionnaires, along with a prepaid reply envelope. Those who did not respond to the letters were contacted by phone.

The EORTC QLQ-C30 questionnaire [18] comprises 30 items and serves as a fundamental tool used across various types of malignancies. It features five functional scales (physical, role, emotional, cognitive, and social), three symptom scales (fatigue, pain, and nausea/vomiting), a global health QoL scale, and six individual items that address common symptoms reported by cancer patients (dyspnea, appetite loss, sleep disturbances, constipation, and diarrhea), as well as perceived financial issues.

The Vulva Module-QLQ-VU34 [19,20], which is still under testing in Phase IV, serves as an additional questionnaire module designed to function alongside the QLQ-C30. It encompasses ten multi-item scales to evaluate various conditions, including changes in vulva skin, vulva scarring, vulva swelling, lymphedema in the groin and legs, urgency and leakage for urine and bowels, body image, sexual enjoyment, and sexually related vaginal changes. Additionally, it includes a single-item assessment focused on vulvo-vaginal discharge. The scoring method for the QLQ-VU34 follows the same fundamental principles as the QLQ-C30.

To enhance interpretation, the scales and items from the questionnaires were converted to a 0 to 100 scale following a scoring manual. This transformation standardizes the data, making it easier to understand and compare the results [18]. In the EORTC QLQ-C30, a higher score on the global QoL and functional scales indicates better functioning and improved QoL, whereas higher scores on the item and symptom scales reflect greater levels of disturbance. For the VU34, higher scores indicate more severe symptoms and poorer functioning, except for the scales measuring sexual activity and sexual satisfaction, where higher scores signify better outcomes. All statistical results are presented as raw numbers and percentages and summarized as means (standard deviation), along with the 95% confidence interval of the mean score.

## 3. Results

### 3.1. Patient Characteristics and Survey Results

Between January 2013 and January 2025, 146 patients underwent surgery for vulvar cancer at the First Obstetrics and Gynecology Clinic of Târgu Mureș, Romania. Of these, 91 met the inclusion criteria for the study. The mean age of patients was 68 years (ranging from 38 to 91); 51.6% were over 70 years old at the time of surgery, with a predominance of 63.73% from rural areas. Histological examination revealed that 86.8% of patients had squamous cell carcinoma of the vulva, while 9.8% presented with other types. Additionally, 48.3% had synchronous VIN III lesions on the final pathological report. Tumor staging according to FIGO 2021 was as follows: IA (4.4%), IB (40.6%), II (14.2%), IIIA (15.3%), IIIB (5.4%), IIIC (15.3%), and IVA (4.4%). Tumors were also grouped according to size: 1 cm (3.3%), 2 cm (16.4%), 3 cm (15.3%), 4 cm (26.3%), and 5 cm (28.5%). Regarding tumor differentiation, 30% were grade 1 (well-differentiated), 38.4% grade 2 (moderately differentiated), and 30.7% grade 3 (poorly differentiated). The depth of stromal invasion indicated that 85.7% of patients had an invasion depth greater than 1 mm, while only 12% had a depth of 1 mm or less. Additional significant findings included positive LVSI in 43.9% of cases and positive resection margins in 30.7% (Table 1 and Table 2).

One of the most significant negative prognostic variables was LN metastases, which was found in 30.7% of patients (Table 2). The two most frequent postoperative complications were wound dehiscence and lymphedema (Table 2). Though, no significant intraoperative complications were reported.

In terms of treatment, 13.1% of patients underwent surgery alone, 18.6% underwent wide local excision, 37.2% underwent hemivulvectomy (anterior, posterior, left or right), 41.7% underwent total radical vulvectomy, and 3.3% underwent PE. Concerning adjuvant therapy, 84.6% received adjuvant RT.

**Figure 2:**
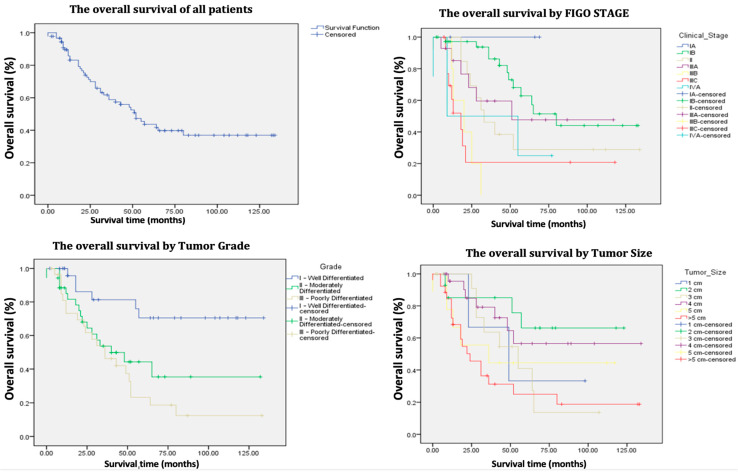
Kaplan–Meier survival curves of the 91 patients treated for vulvar cancer.

**Figure 3:**
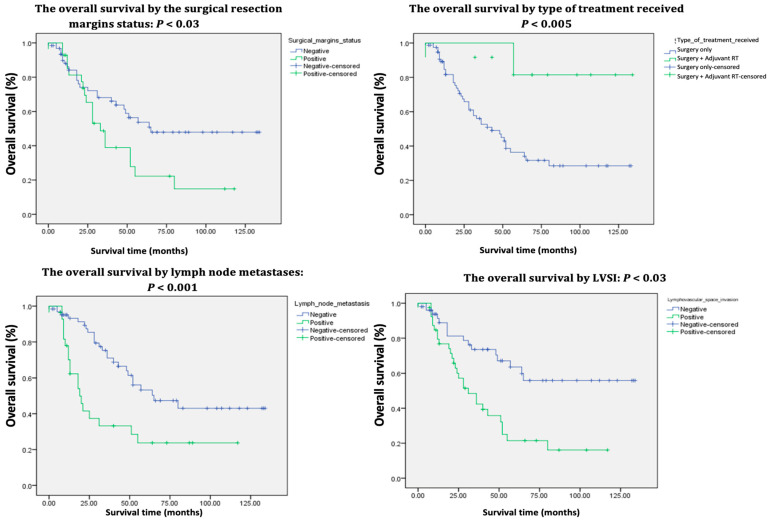
Kaplan–Meier survival curves of the 91 patients treated for vulvar cancer (continued).

The average follow-up period for patients extended up to January 2025 and was 41.9 months (ranging from 0 to 134 months). At the time of assessment, 51.6% of the patients were alive, with a 5-year OS of 45% (Table 2, Figure 2 and Figure 3). Among the survivors, 85.1% were alive without disease, while 14.8% were alive with disease.

#### 3.1.1. Univariate Cox Analysis (Table 1 and Table 2)

The univariate Cox analysis revealed that patients aged 50 years or younger had a significantly lower probability of death (*p* 0.03) and longer survival time, whereas those older than 80 years were associated with a significantly higher probability of death and shorter survival time (*p* = 0.03).

Furthermore, for each categorical increase in age (from 50 years or younger to 51–60, 61–70, 71–80, and >80 years), the risk of death increases by 1.599 times, which is statistically significant (*p* 0.002).

Regarding FIGO stage, each incremental increase in stage corresponds to a 1.373-fold increase in the risk of death, which is statistically significant (*p* 0.0001). Specifically, FIGO stages IIIB and IIIC were associated with significantly shorter survival rates (*p* 0.003 and *p* < 0.005, respectively). In contrast, FIGO stage IB demonstrated significantly longer survival compared to the other stages (*p* 0.006).

Moreover, an increase in tumor diameter by each centimeter is also associated with an elevated risk of mortality and poorer survival outcomes (*p* 0.01), with the worst survival rates linked to tumors larger than 5 cm (*p* 0.003).

In terms of tumor grade, grade I (well differentiated) is associated with longer survival (*p* 0.002) compared to grade III (poorly differentiated), which is associated with shorter survival (*p* 0.007).

Additionally, the presence of lymphovascular space invasion, positive resection margins, and lymph node metastasis were associated with a higher probability of mortality and shorter survival times, with *p*-values of 0.001, 0.05, and 0.002, respectively.

Patients undergoing surgery alone exhibited poorer survival rates compared to those who received adjuvant RT postoperatively (*p* 0.01). Moreover, PE was associated with an increased risk of mortality (*p* 0.03).

#### 3.1.2. Multivariate Cox Analysis (Table 3)

In contrast to univariate analysis, which identifies multiple prognostic factors with either negative or positive significance, the multivariate analysis isolates the following favorable prognostic factors associated with a reduced risk of death and increased survival: age ≤ 50, FIGO stage IB, and tumor differentiation grade I—well differentiated, all with *p*-values < 0.05 (Table 3). Conversely, the following independent factors negatively influenced survival: age > 80, FIGO stages IIIB and IIIC, tumor size > 5 cm, positive resection margins, LN metastasis, and PE, all with *p*-values < 0.05 (Table 1, Table 2 and Table 3).

### 3.2. Results of the QoL Study

Of the 47 alive patients, 68% (n = 32) responded to the QoL questionnaires. The mean age of respondents was 66 years (range 40–79), with 75% (n = 24) receiving adjuvant RT following surgery. The patients were surveyed after a median follow-up period of 56.4 months.

#### 3.2.1. EORTC QLQ-C30 Questionnaire

The EORTC QLQ-C30 [18] is a fundamental questionnaire widely utilized across various malignancies. The survivors reported a reasonably positive global QoL score of 65.3 (median). This result reflects the status of the patients during the week in which they were evaluated.

**Figure 4:**
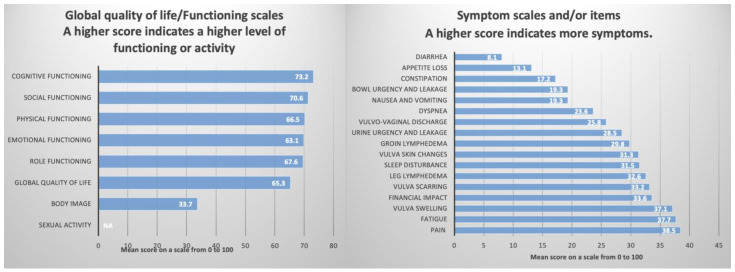
The QoL of the 32 patients who answered the EORTC QLQ-C30 and EORTC QLQ-VU34.

The functional scales assessing physical functioning, role functioning, cognitive functioning, emotional functioning, and social functioning also yielded satisfactory scores, in accordance with the overall QoL (Table 4, Figure 4).

An important section of this questionnaire pertains to the assessment of symptoms experienced by the surveyed patients. The results for nausea, vomiting, dyspnea, appetite loss, constipation, and diarrhea were 19.3%, 23.6%, 13.1%, 17.2%, and 8.1%, respectively, indicating that these symptoms are not of significant concern. However, several symptoms and items reported had a detrimental impact on the QoL. Specifically, fatigue, pain, sleep disturbances, and financial impact were associated with scores of 37.7%, 38.5%, 31.5%, and 33.6%, respectively (Table 4, Figure 4).

#### 3.2.2. EORTC QLQ-VU34 Questionnaire

The EORTC QLQ-VU34 [19,20] must be used in conjunction with QLQ-C30 and is designed to evaluate the specific symptoms associated with vulvar cancer. The results indicate that treatments received negatively impacted the QoL and self-image of these patients, as evidenced by a body image score of only 33.7. Furthermore, there was a complete lack of reported sexual activity, with questions 60–64 left unanswered. This may be attributable to the average age of the surveyed patients (66 years). Regarding the specific symptoms of vulvar cancer, the most concerning values were noted for vulvar swelling, vulvar scarring, leg lymphedema, and vulvar skin changes, all exceeding a score of 30.0 (Table 4 and Figure 4).

## 4. Discussion

Vulvar cancer is considered a rare condition; however, its incidence has been rising in recent decades, particularly among women under the age of 60 [22]. Radical surgery, particularly en bloc resection, has long been considered the primary therapeutic option despite its high complication rates and potential for significant disfigurement [23]. Taussing and Way [24] pioneered the radical vulvectomy technique, which included en bloc bilateral inguinal–femoral lymphadenectomy. Subsequently, the modified radical vulvectomy was introduced, focusing on extensive excision of the primary tumor. Over the years, the significance of inguinal–femoral lymphadenectomy—whether unilateral or bilateral—has evolved, especially with the introduction of SLN biopsy as a less invasive method for LN staging in patients with unifocal tumors under 4 cm and no suspicious groin nodes [23]. Treatment decisions for advanced primary tumors are challenging due to an inconsistent application of chemo/radiotherapy protocols for the vulva, groin, and pelvis, which vary internationally and between institutions [25]. Addressing locally advanced tumors with only radical vulvar excision often results in significant morbidity. Likewise, treatment strategies for advanced vulvar cancer are highly variable, influenced by the infrequency of cases at individual centers and continuous advancements in both surgical and radiation techniques, leaving the efficacy of these treatments somewhat ambiguous [26]. In regard to recurrences, according to the 2024 NCCN Guidelines [21] and 2023 ESGO Guidelines [4], the use of imaging techniques to identify asymptomatic distant metastases has not been shown to provide any clear benefits and is not expected to enhance survival rates, particularly due to the unfavorable prognosis and limited effectiveness of salvage treatments in patients experiencing distant recurrences [22]. Nevertheless, two months post-definitive chemoradiotherapy, at least a CT or a PET/CT should be conducted to confirm complete remission [4,21]. For vulvar recurrences, radical local excision combined with inguinofemoral lymphadenectomy is advised if no prior surgery or only SLN biopsy was performed. Adjuvant therapy indications mirror those for primary disease treatment. If surgery is not an option, external beam RT (EBRT), with or without brachytherapy or concurrent chemotherapy, is recommended [4,21]. In nodal recurrence, radical excision is preferred, with EBRT following surgery in previously untreated patients, while definitive chemoradiotherapy is suitable when surgical intervention is not viable [4,21,22].

In this study, the surgical procedures included wide local resection (18.6%), a type of hemivulvectomy (anterior, posterior, left or right) (37.2%), total radical vulvectomy (41.7%), and PE (3.3%), with a total of 84.6% of patients subsequently receiving adjuvant RT. Additionally, 68.1% of patients underwent LN dissection, with 83.8% receiving bilateral inguinal–femoral lymphadenectomy and 16.1% undergoing unilateral lymphadenectomy, consistent with findings reported by Mansouri et al. [27]. Within the entire cohort of patients who underwent surgery for vulvar cancer, additional neoadjuvant or adjuvant treatments were identified. However, to avoid biased results due to the limited number of these cases, they were excluded from the study.

### 4.1. Prognostic Factors and Survival

Broadly, the 5-year OS of patients with primary vulvar cancer varies between 40% and 90%, whereas the OS for patients experiencing recurrences falls between 25% and 50% [1,4,7,9,12,15,17,22,27,28,29,30,31,32,33,34,35,36]. In the current study, 47 out of 91 patients (51.6%) were alive at the time of investigation, indicating a 5-year OS of 45% and a median survival time of 43 months. These results, while slightly less robust, are comparable to those reported in the earlier referenced studies (Table 1 and Table 2) (Figure 2 and Figure 3). Nevertheless, it is important to note that in the authors’ cohort of 91 patients, 47 (51.64%) were over 70 years old at the time of surgery. Consequently, the 5-year OS of 45% could not be attributed solely to oncological factors, as most of these patients did not die from recurrences but rather from associated comorbidities.

The univariate analysis reveals that survival was negatively impacted by age, FIGO stage, tumor size, tumor differentiation grade, LVSI, resection margin status, LN metastases, and the type of treatment received, all of which displayed *p*-values < 0.05 (Table 1 and Table 2). Conversely, the multivariate analysis exposes age ≤ 50 (*p* < 0.03), FIGO stage IB (*p* < 0.007), and tumor differentiation grade I (*p <* 0.01) to be linked to a decreased risk of mortality and improved survival rates (Table 3) while age > 80 (*p* < 0.05), FIGO stages IIIB (*p* < 0.01) and IIIC (*p* < 0.06), tumor size > 5 cm (*p* < 0.02), positive resection margins (*p* < 0.03), LN metastasis (*p* < 0.06), and PE (*p* < 0.002) were identified as independent negative prognostic factors. The results of the univariate and multivariate analyses are consistent and comparable with those of other similar studies [7,8,22,27,34,37,38,39,40,41,42,43,44,45].

Patients who received adjuvant RT demonstrated superior survival rates compared to those who underwent only surgical procedures, with a 5-year overall survival (OS) rate of 76% (*p* < 0.005). This finding aligns with the research of Woelber et al. [46] and Matsumoto et al. [33]. In terms of age, our study revealed that the risk of mortality increases with each decade, contrasting with the findings of Miljanovic-Spika et al. [7], who found no statistically significant correlations. Younger women are more likely to engage in regular gynecological check-ups, which allows for earlier detection and treatment of vulvar lesions. In contrast, older women typically present with more advanced stages of the disease [43].

Despite the influence of positive resection margins and tumor size greater than 5 cm on the 5-year OS, the historical consensus remains that nodal status is the most significant independent predictor of survival and recurrence in vulvar cancer [8,47]. Baiocchi et al. [30] report that the 5-year OS for patients with positive LN ranges from 30% to 58.5%, whereas this rate increases to between 64.7% and 90.9% for individuals without LN involvement. These findings corroborate the results of the current study, which revealed a 5-year OS of 82% for patients with negative LN (Table 1). Since 62 patients had clinically involved groin nodes, they underwent full inguinal–femoral lymphadenectomy followed by adjuvant groin RT. While this treatment yielded a good response, it was associated with complications, the most common of which included wound dehiscence, lymphedema, and lymphocysts, as also demonstrated by Swift et al. [26]. The 5-year OS was 70% for those without complications (Table 1).

### 4.2. Quality of Life

As observed, the 5-year OS is 45%, with a median survival time of 43 months. Consequently, as many patients with vulvar cancer succeed in surviving the disease, there is an increase in the time they live with treatment-related long-term complications and sequelae. Therefore, post-treatment QoL should be given careful consideration when counseling women regarding their treatment options. Those who undergo surgery for vulvar cancer are particularly vulnerable to sexual dysfunction [48]. A recent longitudinal study revealed a notable reduction in various QoL scales [49]. In this study, nearly 30% of patients received adjuvant RT, and the authors linked the decline in QoL at least in part to the side effects associated with RT. However, treatment satisfaction and stable psychosexual health may not primarily rely on the specific treatment option chosen; rather, they are likely to be more affected by counseling that is customized to meet the patients’ individual preferences [50].

Thus, for the first time in Romania and Eastern European countries, the QoL of patients who have undergone surgery ± adjuvant RT for vulvar cancer will be presented, evaluated using the two questionnaires EORTC QLQ-C30 and EORTC QLQ-VU34. Unsurprisingly, the comparison of the current results with others is limited by the small number of available studies in the literature and the limited number of patients responding to the questionnaires. Additionally, there is significant heterogeneity regarding the therapeutic protocols applied for vulvar cancer across different countries, as well as variations in patient selection criteria, among other factors.

The patients in this study were surveyed after an average follow-up period of 56.4 months post-treatment. Out of the 47 living patients, 32 responded to the questionnaires. The respondents reported a decent overall QoL of 65.3, which is comparable to the results obtained by Novackova et al. [48], although slightly lower than those reported by Hellinga et al. [51]. The patients surveyed underwent either exclusively surgery (25%) or received adjuvant RT (75%), which further impacted their QoL.

However, symptoms specific to the treatment, including vulvar scarring, vulvar swelling, groin lymphedema, and leg lymphedema, negatively affected QoL. Consequently, functional symptoms such as fatigue, pain, and sleep disturbances persisted at an average follow-up of 56.4 months after treatment, leading to a body image perception score of only 33.7 on a scale from 0 to 100 (Table 4). Overall, the results were anticipated and aligned with findings from other studies [48,51,52,53,54,55].

Similarly to the results of Novackova et al. [48], no patients responded to questions regarding sexual functioning (Table 4) (Figure 4). This lack of response may be attributed to the average age of the responding patients (66 years), the delicate and intimate nature of the questions, the absence of a partner, or other reasons. In the study by Trott et al. [52], responses regarding sexual functioning were obtained from only 18.6% of patients, while Oonk et al. [56] received responses from 17.7% and Melo Ferreira et al. [53] from 21.4%. Common among all these studies is the finding that both QoL and sexual functioning are significantly influenced by the type of treatment administered and the average age of the patients. The implementation of targeted physical therapy interventions can play a crucial role in the effective management of lymphedema, helping to reduce swelling, improve limb function, and enhance patients’ overall QoL. Additionally, addressing psychological well-being through supportive counseling or body image therapy is essential, as it can help patients cope with emotional and psychological challenges. Integrating these supportive measures into the multidisciplinary care approach may significantly improve both physical and mental health outcomes for patients [57].

### 4.3. Strength and Limitations

Vulvar cancer is a rare disease. Although there are substantial studies on this topic in Central, Western, and Northern Europe, as well as in the USA and China, similar research is notably lacking in Romania and the broader Eastern European region. The only comparable study in this area was conducted in Croatia in 2021 by Miljanovic-Spika et al. [7], which focused on prognostic factors in vulvar cancer but did not assess patients’ QoL.

Thus, the originality of this study lies in its uniqueness both in Romania and across Eastern European countries. Despite the heterogeneity of the study group and the treatment administered, the analysis effectively provides a description of QoL that is comparable to findings from other studies [48,49,52,53,54,55,56]. Additionally, the identified prognostic factors align with results from similar research [1,4,7,9,12,15,17,22,27,28,29,30,31,32,33,34,35,36].

The heterogeneity of the patient population and treatments, along with the lack of stratification by surgical procedure, comorbidities, or age, limits the ability to draw definitive conclusions about specific subgroups. Future research should aim to address these limitations through larger, more homogenous cohorts and detailed subgroup analyses to provide more nuanced insights.

Nevertheless, the limitations of this study primarily stem from its cross-sectional retrospective observational nature, the heterogeneity of the patient group analyzed, and the different treatments administered. Additionally, the precise timing of disease recurrence could not be accurately determined, which hampered a thorough examination, although this would certainly be of significant interest. Furthermore, the QoL study lacks a control group, such as healthy patients, and does not include a pre-treatment assessment of QoL. Moreover, the analysis did not differentiate QoL outcomes based on the type of treatment received.

## 5. Conclusions

The VULCAN study [8] is acknowledged as the only large multicentric European study focused on evaluating prognostic factors in patients with vulvar cancer. Notably, Romania was not included in its analysis, despite the country’s lack of comprehensive statistical data on the disease.

In the current study, 47 out of 91 patients (51.6%) were alive at the time of investigation, reflecting a 5-year OS of 45% and a median survival time of 43 months. These results, though slightly lower, are comparable to other similar studies [1,4,7,9,12,15,17,22,27,28,29,30,31,32,33,34,35,36]. However, it is important to note that 51.64% of patients were over 70 years old at the time of surgery. Consequently, the OS rate could not be attributed solely to oncological factors, as most of these patients did not die from recurrences but rather from associated comorbidities.

Regarding QoL, respondents reported an good overall QoL score of 65.3, but treatment-related symptoms, such as vulvar scarring, vulvar swelling, and groin and leg lymphedema, adversely affected QoL.

This study highlights relatively favorable OS and QoL outcomes for patients who have undergone surgery ± adjuvant RT for vulvar cancer. It emphasizes the importance of early disease detection and the necessity of special attention to long-term follow-up care. The authors, who have conducted previous research on QoL [58,59], seek to underscore the critical need to consider QoL factors for not only patients with vulvar cancer but also for all gynecological oncology patients. This issue is particularly relevant in a part of Europe where these patients often do not receive adequate government support. While this study identifies key prognostic factors and highlights the importance of QoL, further research is needed to develop specific, evidence-based strategies for incorporating these findings into patient counseling and individualized follow-up plans. Future studies should focus on translating these insights into practical tools for clinical decision-making to optimize patient care and outcomes.

## Figures and Tables

**Table 1:** The features of the 91 participants involved in the study.

	Number (%) or Median (Range)	Overall Survival					Recurrences	
		5-Year Survival Rate	95% CI	Mean Survival (Months)	Univariable		Number	*p* Value
					HR	*p* Value		
**No. of patients**	**91**						**22**	
**Age (years**)	68 (38–91)				1.599	**0.002**		0.745
≤50	8 (8.79%)	14.00%	5.8–26.7	73.0	0.113	**0.031**	1	
51–60	14 (15.38%)	22.00%	11.5–35.9	37.5	0.521	0.215	1	
61–70	24 (26.37%)	20.00%	10.0–33.7	29.5	1.590	0.145	3	
71–80	38 (41.76%)	42.00%	28.1–56.7	28.0	1.173	0.605	2	
>80	7 (7.69%)	2.00%	0.05–10.6	23.0	2.700	**0.027**	0	
**Provenance**						0.563		0.701
Urban	33 (36.26%)	30.00%	17.8–44.6	28.0			3	
Rural	58 (63.74%)	70.00%	55.3–82.1	34.0			4	
**Clinical Stage (Revised FIGO 2021)**					1.373	**0.0001**		
IA	4 (4.40%)	8.00%	2.2–19.2	38.5	0.046	0.344	0	
IB	37 (40.66%)	56.00%	41.2–70.0	43.0	0.393	**0.006**	0	
II	13 (14.29%)	8.00%	2.2–19.2	33.0	1.361	0.410	0	
IIIA	14 (15.38%)	16.00%	7.1–29.1	31.5	0.867	0.745	3	
IIIB	5 (5.49%)	0.00%	0.00–0.00	20.0	4.177	**0.003**	0	
IIIC	14 (15.38%)	10.00%	3.33–21.8	12.5	2.890	**0.005**	3	
IVA	4 (4.40%)	2.00%	0.05–10.6	32.0	1.725	0.363	1	
**Tumor size**					1.306	**0.010**		
1 cm	3 (3.30%)	2.00%	0.05–10.6	49.0	1.041	0.956	0	
2 cm	15 (16.48%)	22.00%	11.5–35.9	57.0	0.392	0.075	2	
3 cm	14 (15.38%)	16.00%	7.1–29.1	38.0	1.166	0.695	0	
4 cm	24 (26.37%)	34.00%	21.2–48.7	36.0	0.494	0.087	2	
5 cm	9 (9.89%)	8.00%	2.2–19.2	36.0	1.181	0.725	0	
>5 cm	26 (28.57%)	18.00%	8.5–31.4	18.5	2.497	**0.003**	3	
**Histology**								
Squamous Cell Carcinoma	79 (86.81%)	90.00%	78.1–96.6	35.0	0.642	0.257	6	0.999
Other types	9 (9.89%)	6.00%	1.2–16.5	26.5	2.161	0.064	1	0.530
Synchronous VIN III	44 (48.35%)	50.00%	35.5–64.4	41.5	0.568	0.063	5	0.256
**Tumor differentiation grade**					1.976	**0.0006**		0.275
Grade 1 (well-differentiated)	28 (30.77%)	44.00%	29.9–58.7	56.0	0.260	**0.0025**	4	
Grade 2 (moderately differentiated)	35 (38.46%)	38.00%	24.6–52.8	28.0	1.268	0.447	2	
Grade 3 (poorly differentiated)	28 (30.77%)	18.00%	8.5–31.4	33.5	2.255	**0.0073**	1	
**Depth of stromal invasion**					6.345	0.067		0.999
≤1 mm	11 (12.09%)	24.00%	13.0–38.1	13.0			1	
>1 mm	78 (85.71%)	76.00%	61.8–86.9	34.0			6	
**Lymphovascular space invasion**					2.789	**0.001**		0.671
Positive	40 (43.96%)	26.00%	14.6–40.3	26.5			3	
Negative	51 (56.04%)	74.00%	59.6–85.3	43.0			4	

Bold: statistically significant results.

**Table 2:** The features of the 91 participants involved in the study (continued).

	Number (%) or Median (Range)	Overall Survival					Recurrences	
		5-Year Survival Rate	95% CI	Mean Survival (Months)	Univariable		Number	*p* Value
					HR	*p* Value		
**Resection margins status**					**1.92**	**0.05**		**0.671**
Positive	28 (30.77%)	20.00%	10.0–33.7	28.0			3	
Negative	63 (69.23%)	80.00%	66.2–89.9	40.0			4	
**Lymph nodes metastases**					2.561	**0.002**		0.671
Positive	28 (30.77%)	18.00%	8.5–31.4	18.0			3	
Negative	63 (69.23%)	82.00%	68.5–91.4	40.00			4	
Patients with Bilateral inguinal–femoral metastasis			*n* = 20					
Patients with Unilateral inguinal–femoral metastasis			*n* = 6					
Avarage number of metastatic lymph nodes/patient			1.3 (1–8)					
Patients with Bilateral inguinal–femoral lymphadenectomy			*n* = 52 (83.8%)					
Patients with Unilateral inguinal–femoral lymphadenectomy			*n* = 10 (16.1%)					
Avarage number of removed lymph nodes/patient			10.6 (8–35)					
**Complications**						0.602		0.430
Yes	28 (30.77%)	30.00%	17.8–44.6	33.5			1	
No	63 (69.23%)	70.00%	55.3–82.1	32.0			6	
Wound dehiscence		*n* = 18						
Lymphedema		*n* = 10						
Lymphocyst		*n* = 12						
Necrosis		*n* = 3						
Rectoperineal fistula		*n* = 1						
Hemorrhagic complications		*n* = 1						
Death < 30 days		*n* = 2						
**Treatment**								
Surgery only	12 (13.19%)	20.00%	10.0–33.7	69.0	0.173	**0.015**	0	
Wide Local Excision	17 (18.68%)	14.00%	5.8–26.7	32.0	1.170	0.663	1	0.999
Hemivulvectomy (ant., post., left or right)	34 (37.2%)	23.00%	11.5–35.9	18.0	0.664	0.436	3	0.292
Total radical vulvectomy	38 (41.76%)	38.00%	24.6–52.8	49.5	0.807	0.483	3	0.999
Pelvic exenteration	3 (3.30%)	0.00%	0.0–0.0	9.0	3.577	**0.034**	0	
**Adjuvant RT**					5.934	**0.005**		0.999
Yes	77 (84.62%)	76.00%	61.8–86.9	28.0			6	
No	14 (15.38%)	24.00%	13.0–38.1	63.0			1	
**Median follow-up duration (months)**	41.9 (0–134)							
**Status**								
Alive	47 (51.65%)	45.05%	34.6–55.8	43.0			7	
Deceased	44 (48.35%)							
Alive free of disease	40 (85.11%)			43.0		0.600		
Alive with disease	7 (14.89%)			64.0				
**Patients who answered the QoL questionnaires**	32 (68%)						0	

**Table 3:** Multivariate Cox analysis.

Variables	*p*-Value	HR	95.0% CI for OR	
			Lower	Upper
**Age ≤ 50**	**0.03**	0.111	0.014	0.904
Age > 80	0.05	2.011	0.757	5.342
FIGO stage IB	0.007	0.275	0.107	0.710
FIGO stage IIIB	0.01	5.130	1.295	20.315
FIGO stage IIIC	0.006	5.532	1.617	18.923
Grade I—Well Differentiated	0.01	0.200	0.057	0.695
Tumor size > 5 cm	0.02	1.835	0.873	3.859
Positive resection margins status	0.03	0.319	0.319	1.918
Lymph node metastasis	0.006	0.069	0.069	1.042
Pelvic exenteration	0.002	3.019	3.019	25.310

HR: hazard ratio; CI: confidence interval; OR: odds ratio.

**Table 4:** The QoL of the 32 patients who answered the questionnaires.

Number of Patients = 32	Items ~	Mean Score	SD *
**QLQ-C30**			
**Functioning scales α**			
Physical functioning α	1–5	70.2	25.9
Role functioning α	6, 7	69.6	29.1
Cognitive functioning α	20, 25	73.2	24.7
Emotional functioning α	21–24	69.9	27.9
Social functioning α	26, 27	71.4	27.7
Global quality of life α	29, 30	65.3	25.9
**Symptom scales and/or items γ**			
Fatigue γ	10, 12, 18	37.7	30.2
Nausea and vomiting γ	14, 15	19.3	20.4
Pain γ	9, 19	38.5	27.1
Dyspnea γ	8	23.6	29.4
Sleep disturbance γ	11	31.5	33.2
Appetite loss γ	13	13.1	22.3
Constipation γ	16	17.2	19.8
Diarrhea γ	17	8.1	22.2
Financial impact γ	28	33.6	32.1
**QLQ-VU**			
**Functional scales/items α**			
Body image α	48–50	33.7	30.3
Sexual enjoyment α	60, 64	NA	NA
Sexually related vaginal changes α	61–63	NA	NA
**Symptom scales and/or items α**			
Vulva skin changes γ	31–34, 37	31.3	27.1
Vulva scarring γ	35, 36	33.2	29.4
Vulvo-vaginal discharge γ	38	25.8	21.2
Vulva swelling γ	39, 40	37.1	30.7
Groin lymphedema γ	41–43	29.8	25.4
Leg lymphedema γ	44–47	32.6	27.3
Urine urgency and leakage γ	52–55	28.5	25.6
Bowl urgency and leakage γ	57–58	19.3	21.9

* Standard Deviation; ~ Numbers match to the item numbers in the QLQ-C30 and QLQ-VU34; α Scores range from 0 to 100, with a higher score indicating a higher level of functioning; **γ** Scores range from 0 to 100, with a higher score indicating a greater grade of symptoms.

## Data Availability

We will provide our data for the reproducibility of this study in other centers if they are requested.
